# Supplementary material for: Identification of Molecular Fragments in Equilibrium with Polysiloxane Ultrasmall Nanoparticles
Source: Nanomaterials (Basel). 2022 Feb 22;12(5):738. doi: 10.3390/nano12050738 (PMC8912117; doi:10.3390/nano12050738)
Supplement: Supplementary file 1 [file nanomaterials-12-00738-s001.zip › nanomaterials-1603265-supplementary.pdf]

Supporting Information

Identification of Molecular Fragments in Equilibrium with Polysiloxane Ultrasmall Nanoparticles

Paul Rocchi [1,2], Lucie Labied [1,3], Tristan Doussineau [2], Michel Julien [2], Barbara Giroud [3], Emmanuelle Vulliet [3], Jérôme Randon [3], Olivier Tillement [1], Agnès Hagège [3] and François Lux [1,4,*]

1 Institut Lumière Matière, Université Claude Bernard Lyon 1, CNRS UMR 5306, 69622 Villeurbanne, France; rocchi@nhtheraguix.com (P.R.); lucie.labied@isa-lyon.fr (L.L.); olivier.tillement@univ-lyon1.fr (O.T.)
2 NH TherAguix S.A, 29 Chemin du Vieux Chêne, 38240 Meylan, France; doussineau@nhtheraguix.com (T.D.); julien@nhtheraguix.com (M.J.)
3 Institut des Sciences Analytiques, CNRS, Université Claude Bernard Lyon 1, Université de Lyon, UMR 5280, 69100 Villeurbanne, France; barbara.giroud@isa-lyon.fr (B.G.); emmanuelle.vulliet@isa-lyon.fr (E.V.); jerome.randon@univ-lyon1.fr (J.R.); agnes.hagege@univ-lyon1.fr (A.H.)
4 Institut Universitaire de France (IUF), 75000 Paris, France
* Correspondence: francois.lux@univ-lyon1.fr; Tel.: +33-(0)4-7243-1200

1. AGuIX
2. Size measurement of AGuIX in water
3. Determination of uncomplexed DOTA amount in AGuIX solution by Cu^{2+} complexation
4. Peak 1 : MS spectra and fragments structures
5. Peak 2 : MS spectra and fragments structures
6. Peak 3 : MS spectra and fragments structures
7. Peak 4 : MS spectra and fragments structures
8. Peak 6 : MS spectra and fragments structures
9. Peak 7 : MS spectra and fragments structures
10. MS spectra comparison: Peak 7 Vs DOTAGA reference

Material & Method

<u>Dynamic Light Scattering (DLS) and ζ-Potential Measurements</u>

Direct measurements of the size distribution of the nanoparticles were performed at 10 g/L via Zetasizer NanoS DLS (Dynamic Light Scattering, laser He-Ne 633 nm) from Malvern Instrument. The ζ-potential of the nanoparticles was also performed via Zetasizer NanoS. Before the measurement, the nanoparticles were diluted to 10 g/L in an aqueous solution containing 0.01 M NaCl.

<u>FTIR Measurement</u>

Approximately 2 mg of the substance to be examined (sample or reference substance) are mixed intimately with about 400 mg of potassium bromide (previously dried 1 hour at 250°C). The mixture is ground, spread uniformly in the suitable die and submitted to a pressure of about 8 tonnes under vacuum during about 10 minutes. The disc must be rejected if visual examination shows lack of uniform transparency or when transmittance at about 2000 cm^{-1} is less than 60%. The measurement is performed through FTIR spectrometer Bruker Invenio-S.

<u>Measurements of Free DOTAGA Groups Based on the Formation of DOTAGA@(Cu^{2+})</u>

The amount of free DOTAGA groups in AGuIX was determined by titration by recording the increase in absorbance at 295 nm due to the formation of DOTAGA@(Cu2+) complex. A series of sample with a fixed amount of product and an increasing amount of Cu2+ was prepared. All the samples were prepared in a pH 4.5 acetate buffer and allowed to react 30 min to ensure a good complexation. Finally, the samples were injected following the HPLC-UV method previously described. The breaks in slope in the absorbance increase were directly related to the amount of uncomplexed DOTAGA in the product.

1. AGuIX synthesis

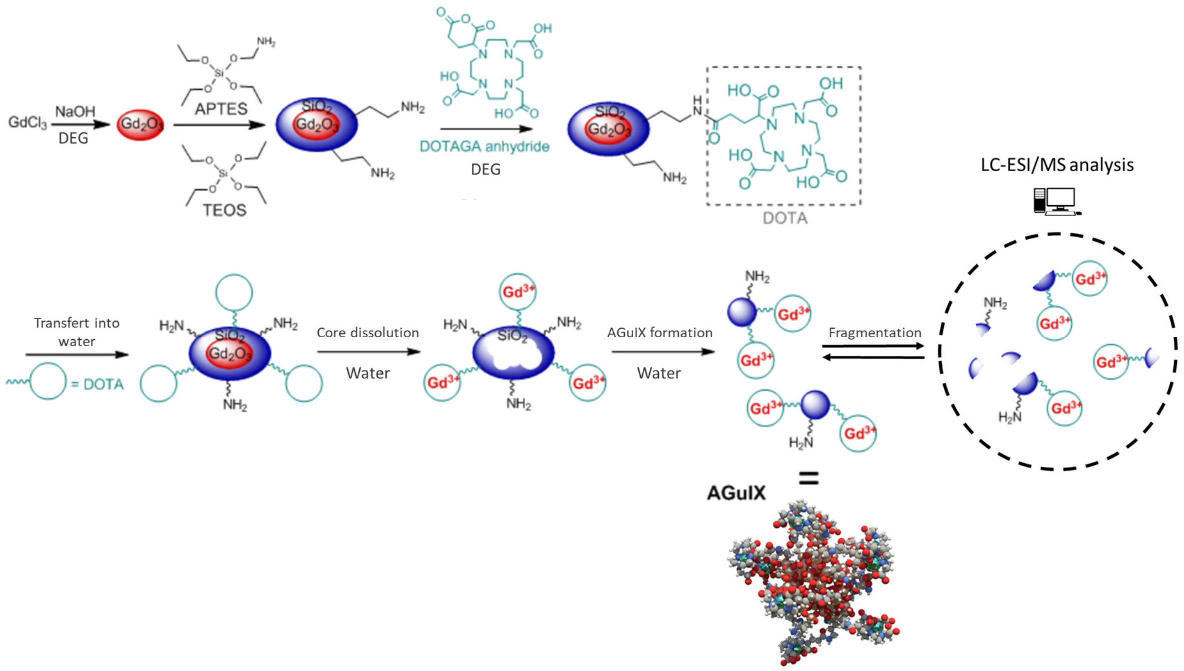

Figure S1. Synthesis route of AGuIX. Adapted from A. Mignot et al., Chem. Eur. J., 2013.

2. Size measurement of AGuIX in water

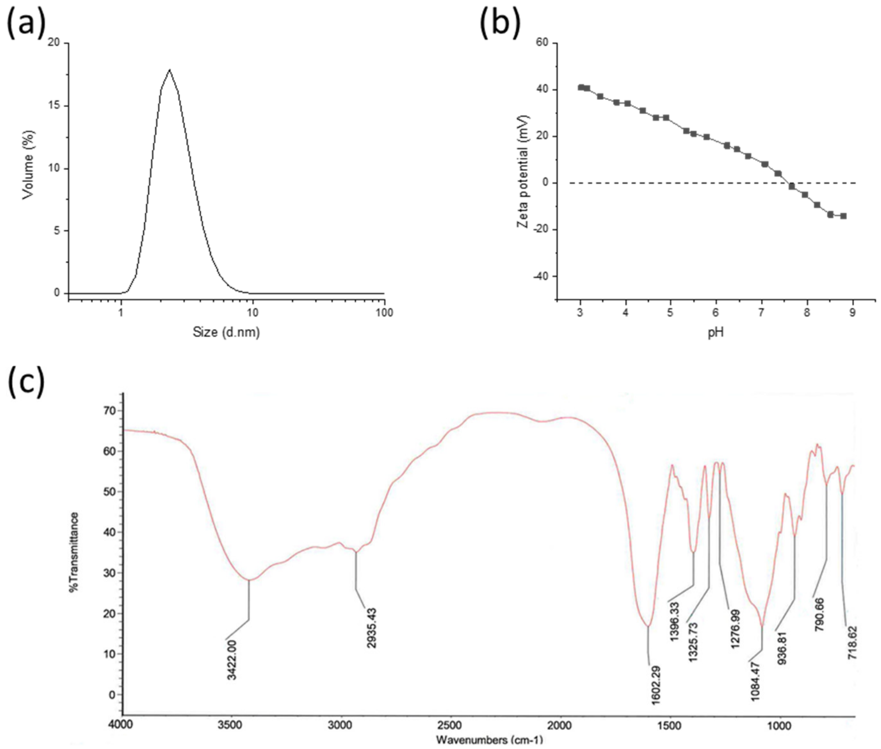

Figure S2. AGuIX main characterizations : (**a**) Hydrodynamic diameter distribution in volum for AGuIX obtained by dynamic light scattering (**b**) Zeta potential vs pH for AGuIX (**c**) FTIR of AGuIX mixed with KBr.

3. Determination of uncomplexed DOTAGA amount in AGuIX solution by Cu^{2+} complexation

In order to evaluate the amount of uncomplexed DOTAGA groups in AGuIX, we performed a titration experiment based on the high absorbance of the complex DOTAGA@Cu^{2+} at 295 nm. We had an increasing amount of Cu^{2+} ions to solutions with a fixed amount of AGuIX. The slope break following the absorbance at 295 nm is directly related to the amount of uncomplexed DOTAGA in the AGuIX solution. Most of the DOTAGA groups in AGuIX are filled by Gd^{3+} ions ([Gd] ≈ 13%w) about 1-2% of the total DOTA amount is ready to chelate other cations like Cu^{2+} as we can see on Figure S3.

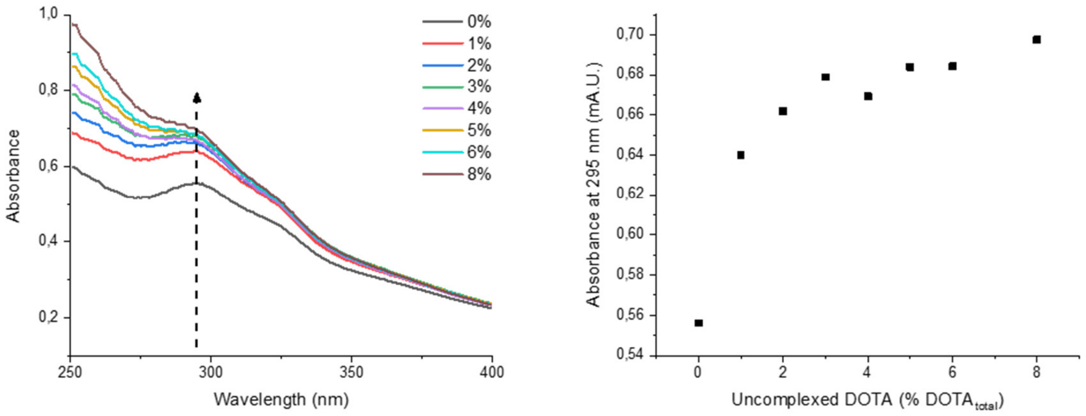

Figure S3. Quantification of uncomplexed DOTAGA. UV spectra of an AGuIX solution and absorbance increase at 295 nm after addition of Cu^{2+} ions in an AGuIX solution. The addition is due to the formation of DOTA@Cu complexes. The slope break is therefore related to the amount of uncomplexed DOTA groups in the mix.

4. Peak 1 : MS spectra and fragments structures

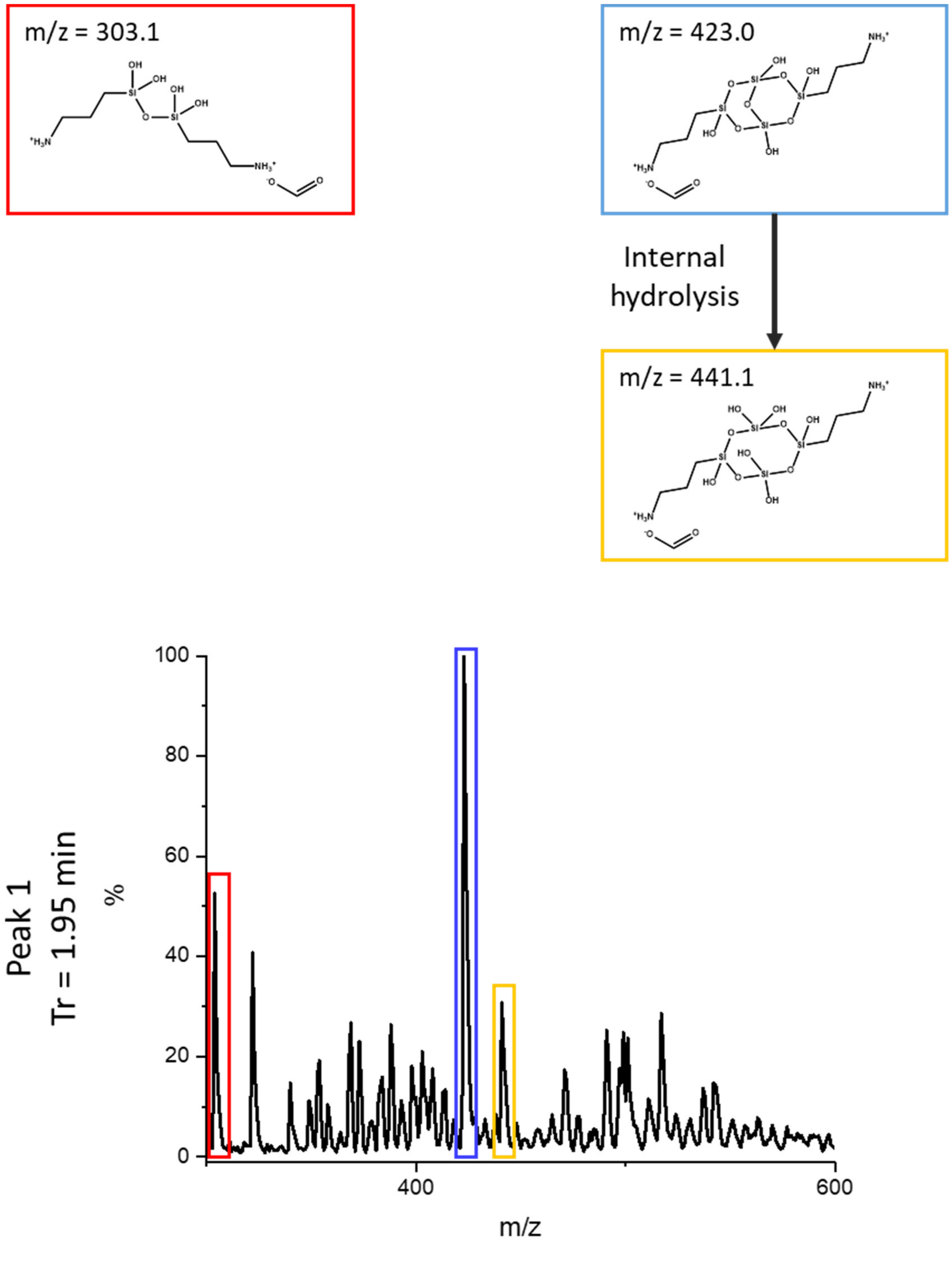

Figure S4. Chemical structures of the fragments identified at HPLC peak 1 (Tr = 1.95 min) and their corresponding m/z signals on the related MS spectra.

5. Peak 2 : MS spectra and fragments structures

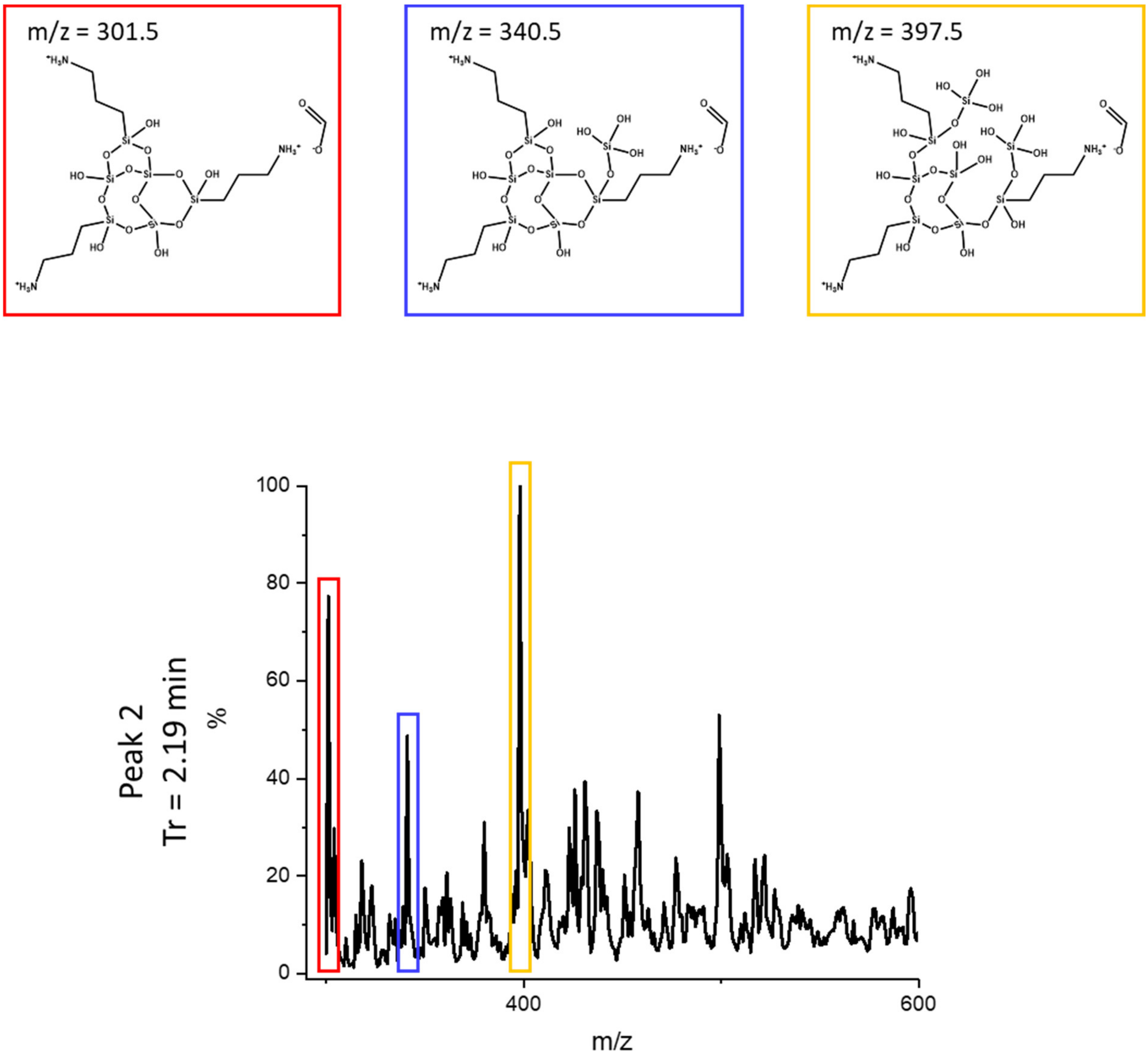

Figure S5. Chemical structures of the fragments identified at HPLC peak 2 (Tr = 2.19 min) and their corresponding m/z signals on the related MS spectra.

6. Peak 3 : MS spectra and fragments structures

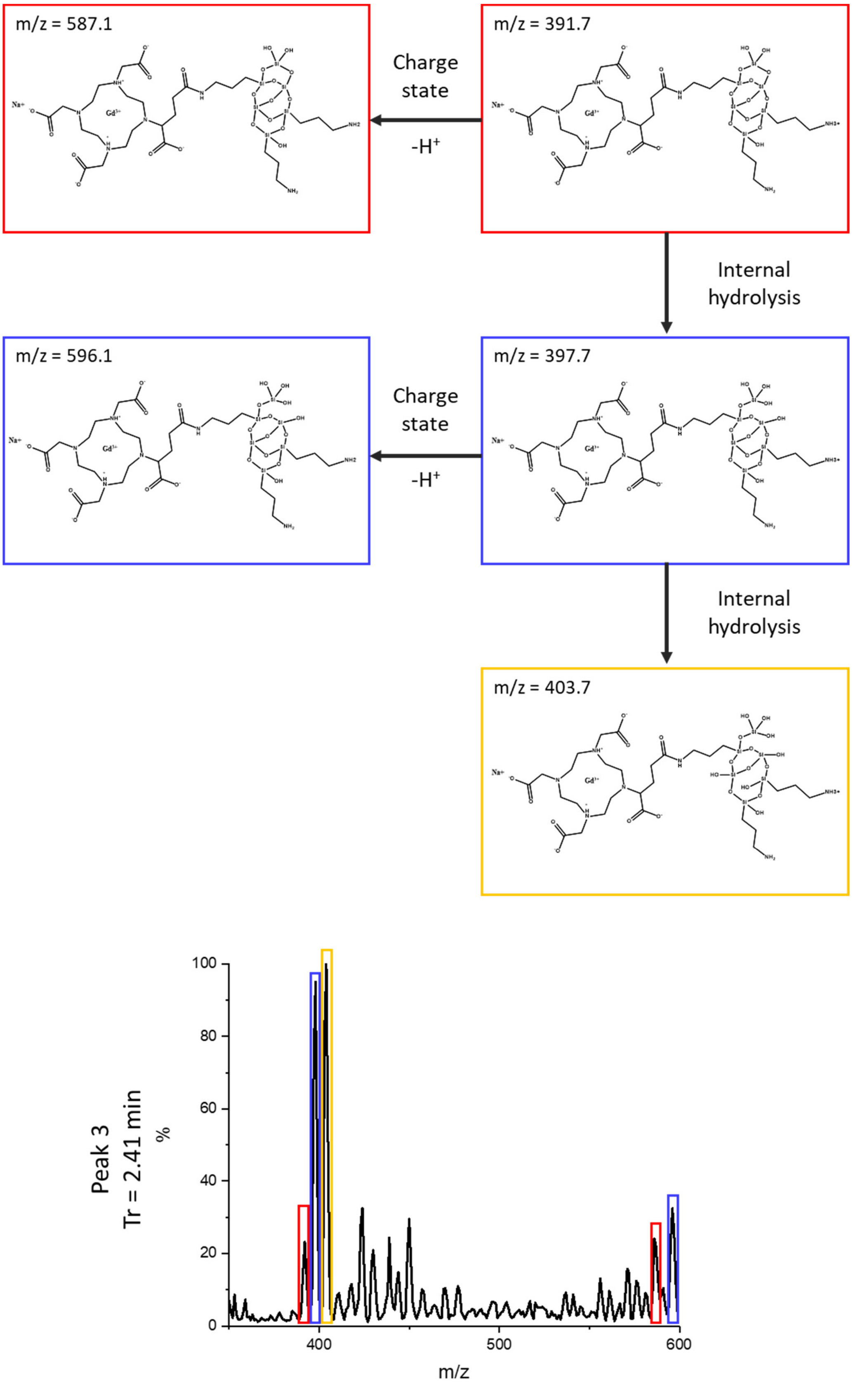

Figure S6. Chemical structures of the fragments identified at HPLC peak 3 (Tr = 2.41 min) and their corresponding m/z signals on the related MS spectra.

7. Peak 4 : MS spectra and fragments structures

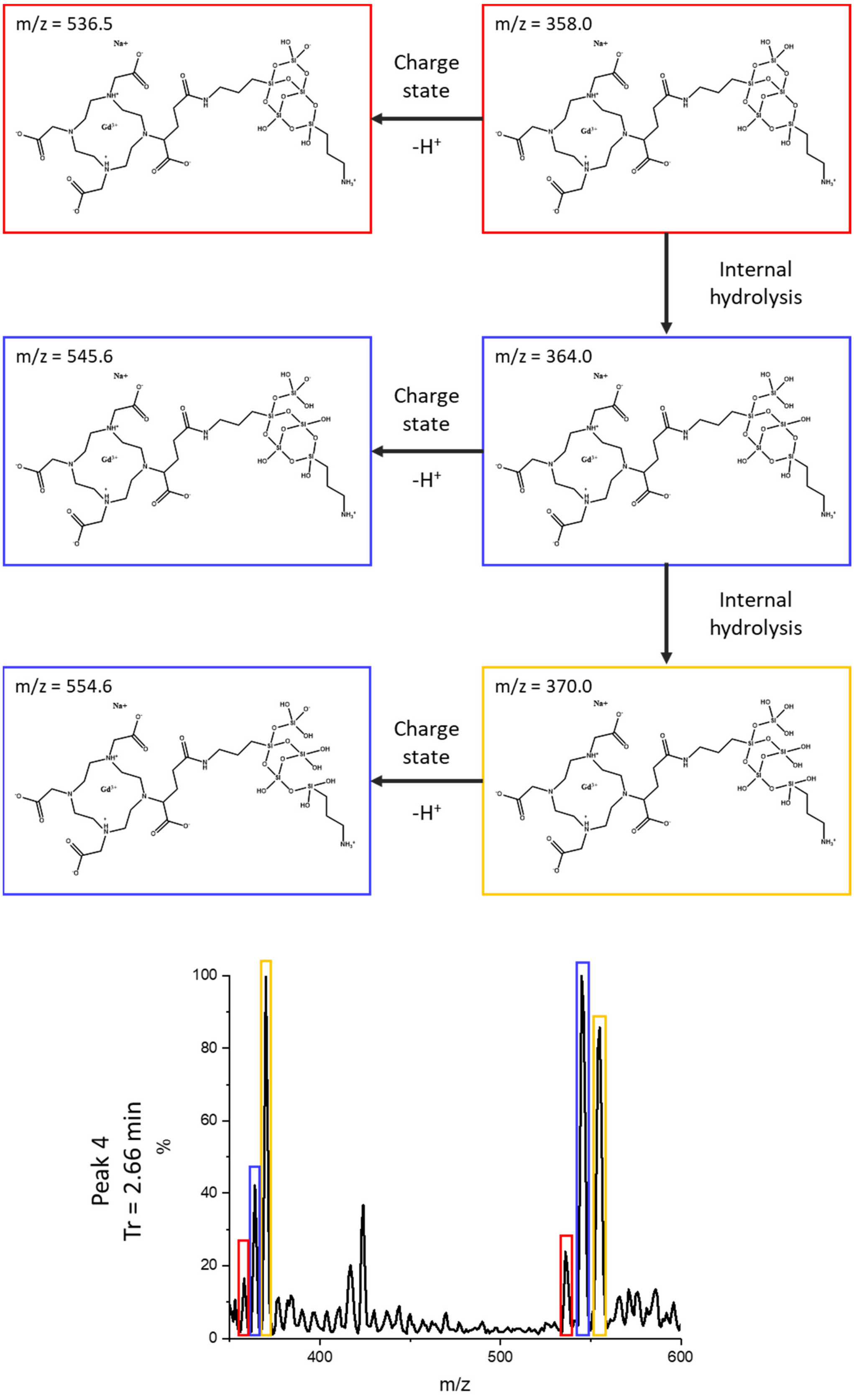

Figure S7. Chemical structures of the fragments identified at peak 4 (Tr = 2.66 min) and their corresponding m/z signals on the related MS spectra.

8. Peak 6 : MS spectra and fragments structures

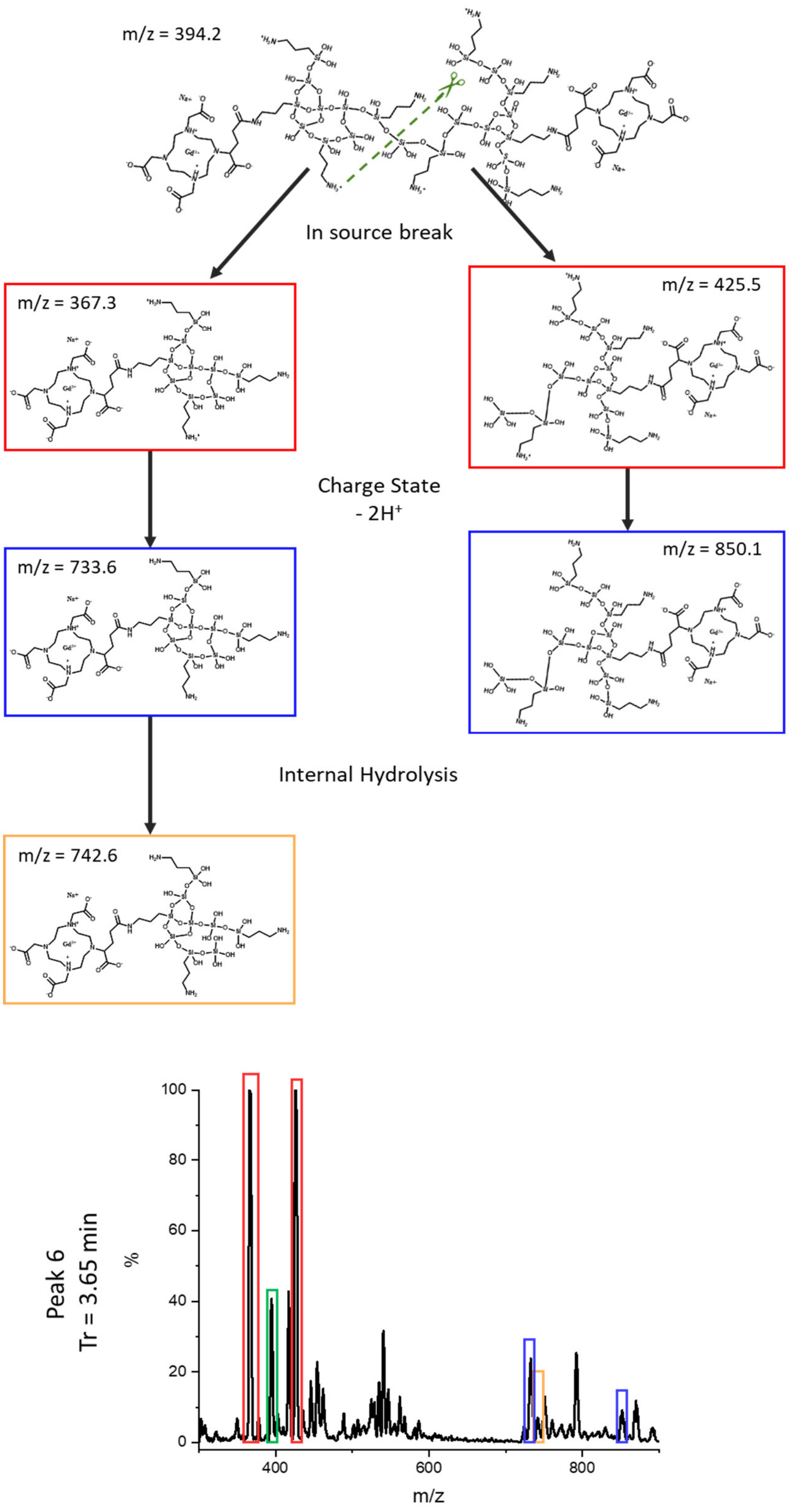

Figure S8. Chemical structures of the fragments identified at HPLC peak 6 (Tr = 3.65 min) and their corresponding m/z signals on the related MS spectra.

9. Peak 7 : MS spectra and fragments structures

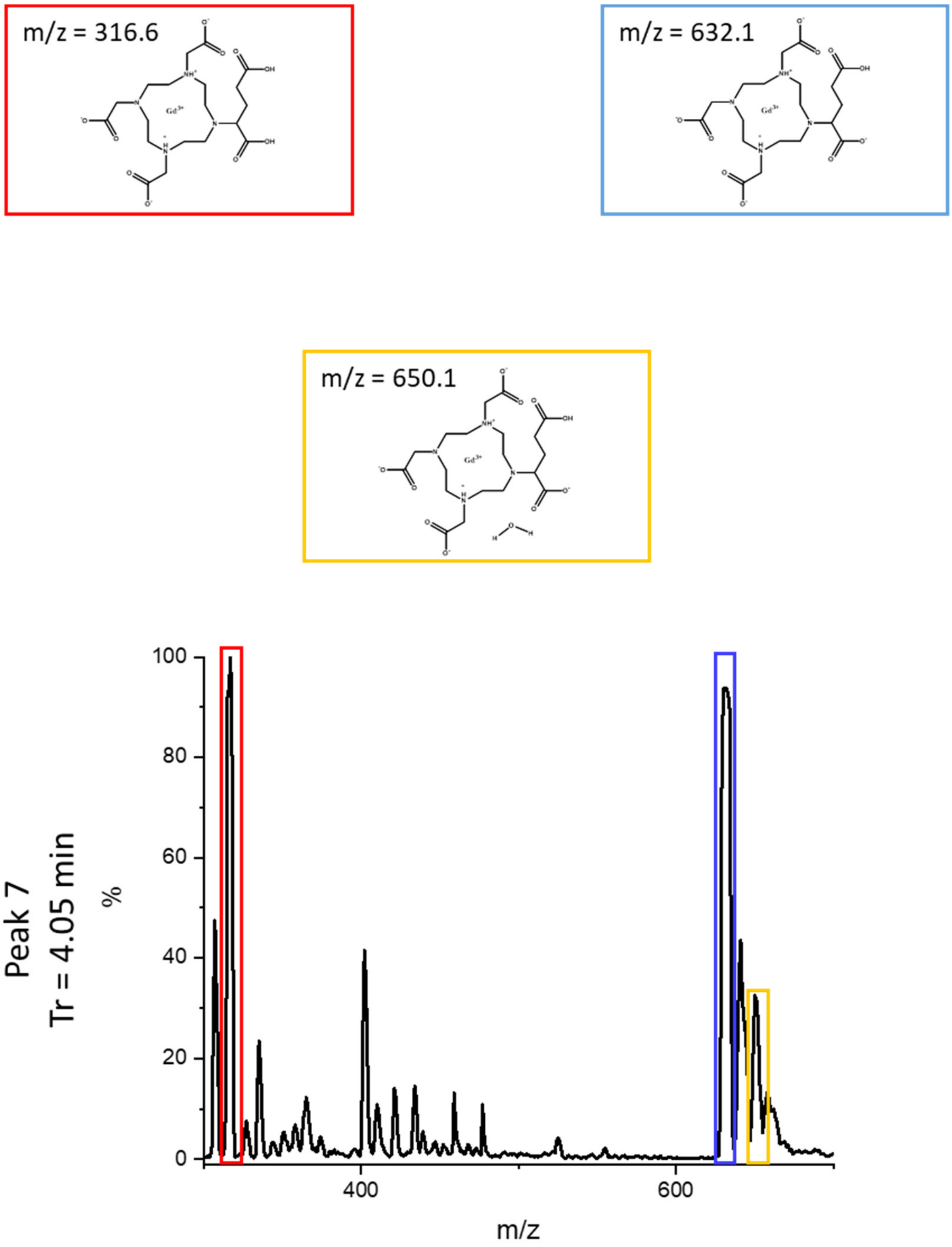

Figure S9. Chemical structures of the fragments identified at peak 7 (Tr = 4.05 min) and their corresponding m/z signals on the related MS spectra.

10. MS spectra comparison: Peak 7 Vs DOTAGA(Gd) reference

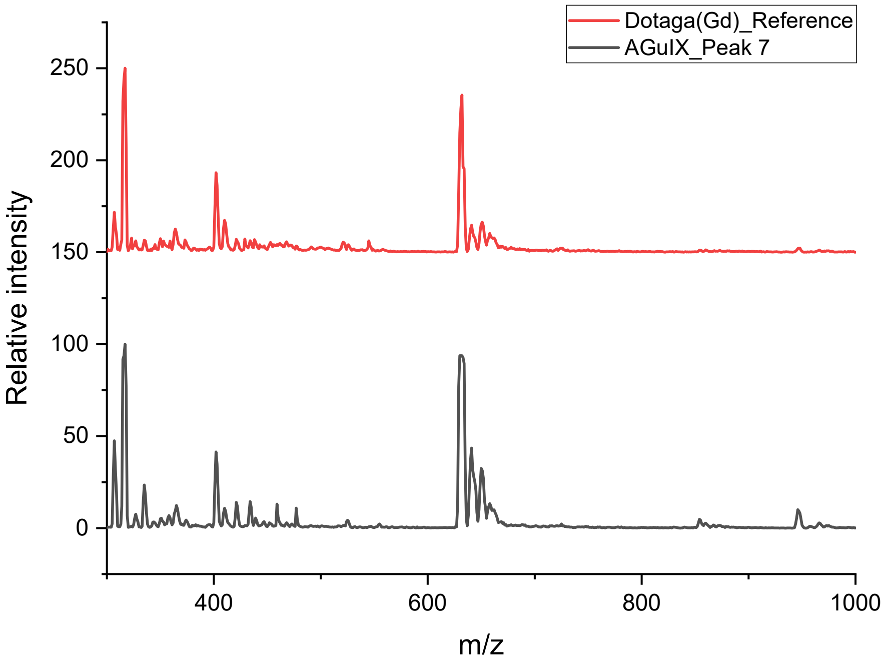

Figure S10. MS spectra comparison of HPLC peak 7 (Tr = 4.05 min) and DOTAGA(Gd) control sample.
